# Trimethylamine N-Oxide Concentration and Blood Pressure in Young Healthy Men and Women: A Replicated Crossover Study

**DOI:** 10.3390/metabo13070876

**Published:** 2023-07-24

**Authors:** Samantha N. Rowland, Liam M. Heaney, Mariasole Da Boit, Stephen J. Bailey

**Affiliations:** 1 School of Sport, Exercise and Health Sciences, Loughborough University, Loughborough LE11 3TU, UK; s.rowland@lboro.ac.uk (S.N.R.); l.m.heaney2@lboro.ac.uk (L.M.H.); 2 Health and Life Sciences, School of Allied Health Sciences, De Montfort University, Leicester LE1 9BH, UK; mariasole.daboit@dmu.ac.uk

**Keywords:** gut metabolite, liquid chromatography–tandem mass spectrometry, cardiovascular health, menstrual cycle, hormonal contraceptives, biological sex

## Abstract

Trimethylamine N-oxide (TMAO), a gut-derived metabolite and marker of gut dysbiosis, has been linked to hypertension. Blood pressure is proposed to be elevated in hormonal contraceptive users and males compared to age-matched eumenorrheic females, but the extent to which TMAO differs between these populations has yet to be investigated. Peripheral and central blood pressure were measured, with the latter determined via applanation tonometry, and plasma TMAO concentration was assessed using liquid chromatography–tandem mass spectrometry. The following variables were assessed on two occasions in each of the following conditions: the early follicular phase (EFP) and mid-luteal phase (MLP) in eumenorrheic women (*n* = 13), and the pill-free interval (INACTIVE) and pill consumption days (ACTIVE) in women using oral contraceptive pills (*n* = 12), and in men (*n* = 22). Briefly, 17-β-estradiol and progesterone concentrations were quantified via ELISA in all females. There were no differences in TMAO concentration between EFP (2.9 ± 1.7 μmol/L) and MLP (3.2 ± 1.1 μmol/L), between INACTIVE (3.3 ± 2.9 μmol/L) and ACTIVE (2.3 ± 1.1 μmol/L) days, or between men (3.0 ± 1.8 μmol/L), eumenorrheic women (3.0 ± 1.3 μmol/L) and contraceptive users (2.8 ± 1.4 μmol/L). Blood pressure was consistent across the menstrual cycle and pill days, but brachial systolic blood pressure was higher in males than females. There were no differences in brachial diastolic blood pressure or central blood pressure between the sexes. Repeated measures of TMAO, blood pressure, 17-β-estradiol and progesterone were consistent in all populations. These findings suggest that the link between TMAO and blood pressure is limited in healthy young adults.

## 1. Introduction

The role of the intestinal microbiome in health and disease has garnered significant interest with recent research revealing sexual dimorphisms in the gut microbiome [1]. As such, sex differences in the composition of the microbiome has been postulated as an explanation for inter-sex differences in vascular risk factors [2,3]. Evidence to support the role of the gut microbiome in the aetiology of cardiovascular disease is provided by the positive association between Trimethylamine N-oxide (TMAO) [4], a gut-derived metabolite and marker of gut dysbiosis, and hypertension [5]. TMAO is formed from micronutrients such as choline, betaine and L-carnitine (present in foods such as red meat, eggs, fish and seafood), via gut microbial metabolism [6,7,8,9]. Microbes residing in the large intestine can transform these dietary precursor compounds into Trimethylamine (TMA), which is subsequently oxidised by hepatic enzyme Flavin-containing monooxygenase 3 (FMO3) to form TMAO [10].

It has been postulated that men may possess protective physiological mechanisms with respect to endogenous TMAO formation [3]. However, some [11,12,13], but not all [14,15] previous studies have reported higher TMAO concentrations in men than women. Moreover, TMAO has been positively associated with blood pressure [16] and, when matched for age, young healthy females typically exhibit lower blood pressure than males do [17]. However, a significant proportion of the female population elect to use oral contraceptive pills to reduce menstrual symptoms, allow for cycle manipulation and prevent pregnancy, which downregulates endogenous concentrations of 17-β-estradiol and progesterone [18]. Oral contraceptive use is commonly associated with mild increases in blood pressure [19,20], but the extent to which TMAO levels differ between populations with a distinct hormonal milieu has yet to be investigated. Moreover, there has been limited interrogation of TMAO levels across different phases of the menstrual cycle, and no investigation of TMAO concentrations on pill consumption and pill withdrawal days in contraceptive users. This is of importance given the proposed link between TMAO and cardiovascular risk [21], and the heightened predisposition for elevated blood pressure in both young males and females using oral hormonal contraceptives compared with eumenorrheic females.

There is an absence of research exploring the reproducibility of circulating TMAO, 17-β-estradiol and progesterone hormone data, and blood pressure when measured on two separate occasions under the same control conditions. Replicate control studies are sparse within some scientific fields due to their time-consuming nature, but are needed to elucidate true response heterogeneity to interventions (such as the administration of nutritional supplements) from random within-participant measurement variability [22]. At present, there is a substantial body of literature reporting inter-individual variability in response to, for example, exercise and nutritional interventions; however, many of these studies are not appropriately designed to interrogate such variability and do not account for random biological and behavioural fluctuations [23]. Thus, further research is needed to determine random within-participant variations and measurement errors in commonly used techniques/protocols to better understand the presence or absence of responders and non-responders in intervention studies.

The aim of this study was two-fold. Firstly, to assess whether or not TMAO and blood pressure differ across the menstrual cycle, between pill consumption and pill withdrawal days in contraceptive pill users, and in males versus females. Secondly, to assess the reproducibility of TMAO, brachial and central blood pressure, 17-β-estradiol and progesterone under basal conditions.

## 2. Materials and Methods

### 2.1. Participants

Briefly, 13 eumenorrheic women (mean ± SD, age: 23 ± 5 years; stature: 1.67 ± 0.06 m; body mass: 64.9 ± 9.3 kg; BMI: 23.4 ± 3.7 kg^.^m^2^), 12 women taking oral contraceptive pills (age: 22 ± 4 years; stature: 1.64 ± 0.08 m; body mass: 63.3 ± 8.7 kg; BMI: 23.4 ± 2.6 kg^.^m^2^), and 23 men (age: 23 ± 3 years; stature: 1.80 ± 0.08 m; body mass: 80.4 ± 11.5 kg; BMI: 24.8 ± 2.3 kg^.^m^2^) volunteered to participate in this study. All participants were classified as being either recreationally active or trained [24] and none were reported as tobacco smokers or had a history of metabolic, digestive, cardiovascular or renal disease. No participants were knowingly pregnant, lactating, trying to become pregnant or using hormone replacement therapy. The study was approved by Loughborough University Research Ethics Approvals Human Participants Sub Committee (ethics code: R19-P137) and participants gave written informed consent prior to participation.

### 2.2. Experimental Design

#### 2.2.1. Eumenorrheic Women

**Figure 1:**
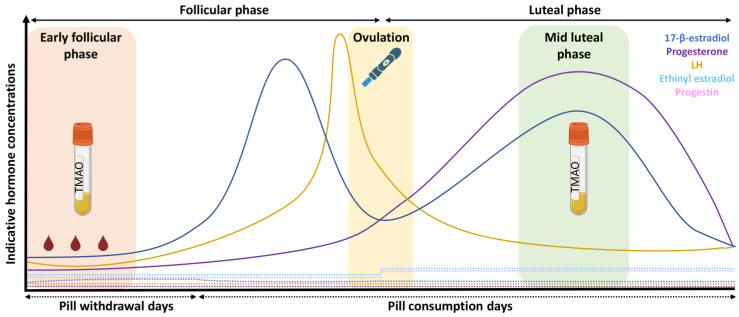
Schematic displaying endogenous (17-β-estradiol, progesterone, and luteinising hormone—LH) hormonal fluctuations throughout the menstrual cycle, with ovulation occurring mid-way in eumenorrheic women (solid lines), and the hormonal profile of combined, monophasic oral contraceptive pill users (dashed lines), including endogenous (17-β-estradiol, progesterone and LH) and exogenous (ethinyl estradiol and progestin) hormone concentrations.

All eumenorrheic women had a regular, natural menstrual cycle (21–35 days in duration, with 9 or more consecutive periods per year and evidence of a luteinising hormone (LH) surge, without the use of any hormonal contraceptives within the last 6 months) [25]. Testing was completed during two separate menstrual cycles and captured two distinct hormonal profiles: (1) the early follicular phase (EFP, days 1–4), i.e., low concentrations of 17-β-estradiol and progesterone; (2) the mid-luteal phase (MLP, the middle four days of the luteal phase, determined from the predicted cycle duration minus the day of ovulation, which was ~7–9 days after a positive urinary LH test), i.e., high concentrations of 17-β-estradiol and progesterone (Figure 1).

In line with recently published methodological recommendations [26], calendar-based counting, urinary LH surge ovulation detection kits and serum measurements of sex hormones, 17-β-estradiol and progesterone were used to plan and verify the timing of testing throughout two menstrual cycles. The EFP was determined via the self-reported onset of menses, with day 1 indicated as the start of menstrual bleeding. Urinary LH test kits (One Step Ovulation Test, Home Health Diagnostics, Watford, UK) were used each morning from 3 days prior to the estimated day of ovulation. Participants inserted test strips into their urine and interpreted the result according to the manufacturer’s instructions, with visual confirmation provided to the researcher to identify the LH surge. Positive test results gave the researchers confidence that ovulation occurred within 14–26 h of the urinary LH peak. Participants were asked to confirm the day of the next onset of menses to verify the test cycle duration and whether or not the mid-luteal window was captured. Both menstrual cycle phases (EFP and MLP) were subsequently confirmed by serum measurements of 17-β-estradiol and progesterone, with a mid-luteal progesterone concentration of ≥16 nmol L^−1^ (i.e., ≥5 ng/mL^−1^) being used to verify ovulation [26].

#### 2.2.2. Oral Contraceptive Pill Users

The inclusion criteria permitted the recruitment of women using hormonal contraception, specifically 21-day, combined, monophasic oral contraceptive pills, for at least 6 months. The brands included Rigevidon^®^ (*n* = 5), Lucette^®^ (*n* = 2), Levest^®^ (*n* = 2), Millinette^®^ (*n* = 1), Cilique^®^ (*n* = 1) and Lizinna^®^ (*n* = 1). Contraceptive users were tested twice during the off-pill phase (INACTIVE) (days 1–4), i.e., when no exogenous synthetic hormones were ingested; and twice during the on-pill phase (ACTIVE) (days 17–21). Days 1–4 were chosen to ensure that endogenous concentrations of 17-β-estradiol and progesterone were low. Days 17–21 were selected as the end of the active pill phase where endogenous concentrations of 17-β-estradiol and progesterone are low but circulating concentrations of the synthetic, ingested hormones ethinyl estradiol and progestin concentrations are slightly elevated (Figure 1). All participants confirmed pill ingestion at similar times each day.

#### 2.2.3. Experimental Procedures

Females completed 4 experimental visits in total. A population of age-matched males were also recruited and completed 2 experimental visits, ≥48 h apart. All visits were completed in a randomised, repeated-measures, crossover experimental design. Prior to each visit, participants were contacted to confirm the absence of COVID-19 symptoms and compliance with pre-test control procedures. Participants were instructed to maintain their habitual exercise patterns for the duration of the study but were required to avoid strenuous exercise and alcohol ingestion in the 24 h prior to each visit. Participants were asked to record their dietary intake 24 h before their first visit and were asked to replicate this before each subsequent visit. All experimental within-participant visits were performed at the same time of day (±1 h).

### 2.3. Measurements

#### 2.3.1. Peripheral Blood Pressure

Participants were required to rest supine for 10 min in a dark room. Thereafter, blood pressure of the brachial artery was measured using an automated sphygmomanometer (Omron Healthcare, Kyoto, Japan). Five measurements were taken and the mean of the five measurements was used for analysis.

#### 2.3.2. Central Blood Pressure

Following 20 min of supine rest, pulse wave analysis was conducted on the radial artery using applanation tonometry methods (SphygmoCor; Atcor Medical, Sydney, Australia). Pulse wave analysis calibrated to brachial blood pressure involves a validated generalised transfer function to derive corresponding central aortic pressures. All tonometry data were recorded by a single investigator, a minimum of two recordings were taken at each time interval and the two measurements with the highest quality index (>80%) were accepted for analysis. Pulse wave analysis indices of interest included aortic systolic and diastolic blood pressure.

#### 2.3.3. Blood Collection

Following 30 min of supine rest, a tourniquet was applied around the upper arm and venous blood was subsequently drawn from an antecubital vein via venepuncture into EDTA and serum vacutainers. Plasma was subsequently extracted and stored at −80 °C for later TMAO determination. The serum tube was left to clot at room temperature before being centrifuged (3500× *g* at 4 °C for 10 min). Serum was then aliquoted into Eppendorf tubes and frozen at −80 **°**C for subsequent 17-β-estradiol and progesterone analysis.

### 2.4. Sample Extraction and Quantification

#### 2.4.1. Materials

All solvents (water, methanol, and acetonitrile) were of LC-MS grade and purchased from Fisher Scientific (Loughborough, UK) or VWR International (Lutterworth, UK). TMAO (98.9% purity) was purchased from Merck (Gillingham, UK) and D_9_-TMAO (>98% purity, 99.9% enrichment) from Cambridge Isotopes (Tewksbury, MA, USA). Formic acid and ammonium hydroxide were purchased from Fisher Scientific.

#### 2.4.2. Plasma TMAO

Plasma TMAO concentraiton was quantified using liquid chromatography–tandem mass spectrometry (LC-MS/MS). Data were processed using MassLynx 4.1 software. An Acquity UPLC liquid chromatograph was coupled to a Quattro Ultima triple-quadrupole mass spectrometer (Waters Corp., Milford, MA, USA) operated in multiple reaction monitoring (MRM) mode. Briefly, 20 μL of the plasma sample was mixed with 80 μL of 10 μmol/L deuterated TMAO (D_9_-TMAO) in methanol. Samples were then vortexed for 10 s and centrifuged at 21,100× *g* for 10 min at 4 °C. The subsequent supernatant was then transferred into vials for analysis.

Chromatographic separation was performed according to a previously validated method [27] using a UPLC BEH HILIC column (130 Å, 1.7 μm, 2.1 mm × 100 mm; Waters Corp., Milford, MA, USA) and pre-column (Acquity VanGuard; 130 Å, 1.7 μm, 2.1 mm × 5 mm; Milford, MA, USA). The sample injection volume was 5 μL. The column was heated to 50 °C and the solvent flow rate was maintained at 600 μL/min. Solvent A was 0.025% ammonium hydroxide and 0.045% formic acid and solvent B was pure acetonitrile. The gradient started at 95% B and reduced linearly to 4% B in 0.8 min before returning to 95% B at 1.9 min until 2.5 min. MRM was performed via electrospray ionisation in positive ionisation mode using precursor ions of *m/z* 76.1 for TMAO and *m/z* 85.1 for D_9_-TMAO, with collision energies at 20 V (TMAO) and 25 V (D_9_-TMAO) to monitor fragment ions of *m/z* 58.1 and *m/z* 66.1, respectively. Calibration standards (0–50 μmol/L) were prepared immediately prior to analysis. Samples were quantified using QuanLynx 4.1 software based on the ratio of peak areas for TMAO to D_9_-TMAO and compared to a calibration curve which was constructed by plotting the peak area ratio of the metabolites to internal standards against the concentrations. Based upon duplicate sample measurements, the intra-assay coefficient of variation (CV) was 3.0%.

#### 2.4.3. Serum 17-β-Estradiol and Progesterone

Briefly 17-β-estradiol and progesterone concentrations were determined in duplicate using competitive immunoenzymatic assays in accordance with the manufacturer’s instructions (Abcam, Cambridge, UK: ab108667 and ab108670, respectively). The intra-assay CV for 17-β-estradiol was 3.6%, with a detection limit of 8.68–2000 pg/mL and 2.4%, with a detection limit of 0.05–40 ng/mL for progesterone.

### 2.5. Statistical Analysis

SPSS version 27 was used for all statistical analysis. Shapiro–Wilk’s test was used to check data normality, and the Greenhouse–Geisser correction was used when sphericity was violated. Two-way repeated-measures ANOVAs were used to analyse the effects of phase (EFP vs. MLP and INACTIVE vs. ACTIVE) × biological repeat (i.e., experimental visit 1 vs. 2 in the same phase) in eumenorrheic women and contraceptive users. Independent-samples *t* tests were used for planned comparisons to compare EFP vs. INACTIVE (i.e., low hormone comparison) and MLP vs. ACTIVE (i.e., ‘high’ hormone comparison). Paired-samples *t* tests were used to assess the reproducibility of biological repeats in males. To compare TMAO and blood pressure variables between eumenorrheic women, contraceptive users and males, data were averaged across the menstrual cycle (eumenorrheic women) and pill days (contraceptive users), and one-way repeated-measures ANOVAs were used. Significant main effects for groups were followed up with post hoc Holm–Bonferroni corrected independent-samples *t* tests. To calculate effect sizes, partial eta squared (*n_p_^2^*) was used for the omnibus tests, Cohen’s d (mean_2_ − mean_1_/SD_pooled_) was used for independent-samples *t* tests and Cohen’s d_z_ (*t*/√n) was used for paired-samples *t* tests. Pearson’s product–moment correlation coefficients were used to assess the relationships between TMAO and blood pressure. To visualise the relationship between TMAO and continuous variables, a simple linear regression analysis was performed using GraphPad Prism 9.5.1. All data are displayed as mean ± SD unless otherwise stated. Statistical significance was accepted at *p* ≤ 0.05.

## 3. Results

### 3.1. TMAO

There were no main effects identified for phase (*n_p_^2^* = 0.06, *n_p_^2^* = 0.09), repeat (*n_p_^2^* = 0.07, *n_p_^2^* = 0.20) or phase × repeat interaction (*n_p_^2^* = 0.01, *n_p_^2^* = 0.03) in eumenorrheic women (Table 1) or contraceptive users (Table 2), respectively. There were no differences between EFP (2.9 ± 1.7 μmol/L) and INACTIVE (3.3 ± 2.9 μmol/L, *p* = 0.693, d = 0.17) or between MLP (3.2 ± 1.1 μmol/L) and ACTIVE (2.3 ± 1.1 μmol/L, *p* = 0.053, d = 0.82). In males, there were no differences between visits (3.7 ± 2.9 μmol/L vs. 2.4 ± 2.3 μmol/L, *p* = 0.146, d_z_ = 0.33). There was no main effect of group (*p* = 0.514, *n_p_^2^* = 0.06) between males, eumenorrheic women and contraceptive users (Table 3).

### 3.2. Peripheral Blood Pressure

#### 3.2.1. Brachial Systolic Blood Pressure

There were no main effects identified for phase (*n_p_^2^* = 0.00, *n_p_^2^* = 0.06), repeat (*n_p_^2^* = 0.00, *n_p_^2^* = 0.15) or phase × repeat interaction (*n_p_^2^* = 0.05, *n_p_^2^* = 0.22) in eumenorrheic women (Table 1) or contraceptive users (Table 2). There were no differences between EFP (106 ± 8 mmHg) and INACTIVE (108 ± 5 mmHg, *p* = 0.491, d = 0.30) or between MLP (106 ± 7 mmHg) and ACTIVE (108 ± 6 mmHg, *p* = 0.291, d = 0.31). In males, there were no differences between visits (119 ± 7 mmHg vs. 119 ± 7 mmHg, *p* = 0.688, d_z_ = 0.09). There was a main effect of group (*p* < 0.001, *n_p_^2^* = 0.68), with brachial systolic blood pressure being higher in males compared to that in eumenorrheic women (d = 1.86) and contraceptive users (d = 1.81), both *p* < 0.001 (Table 3).

#### 3.2.2. Brachial Diastolic Blood Pressure

There were no main effects identified for phase (*n_p_^2^* = 0.01, *n_p_^2^* = 0.17), repeat (*n_p_^2^* = 0.02, *n_p_^2^* = 0.14) or phase × repeat interaction (*n_p_^2^* = 0.00, *n_p_^2^* = 0.01) in eumenorrheic women (Table 1) or contraceptive users (Table 2). There were no differences between EFP (67 ± 7 mmHg) and INACTIVE (68 ± 3 mmHg, *p* = 0.583, d = 0.19) or between MLP (67 ± 6 mmHg) and ACTIVE (68 ± 4 mmHg, *p* = 0.771, d = 0.20). In males, there were no differences between visits (67 ± 5 mmHg vs. 67 ± 5 mmHg, *p* = 0.394, d_z_ = 0.19). There was no main effect of group (*p* = 0.765, *n_p_^2^* = 0.02) between males, eumenorrheic women and contraceptive users (Table 3).

### 3.3. Central Blood Pressure

#### 3.3.1. Aortic Systolic Blood Pressure

There were no main effects identified for phase (*n_p_^2^* = 0.01) or repeat (*n_p_^2^* = 0.02), but there was a phase × repeat interaction (*n_p_^2^* = 0.36) in eumenorrheic women (Table 1). There were no main effects identified for phase (*n_p_^2^* = 0.04), repeat (*n_p_^2^* = 0.13), or phase × repeat interaction (*n_p_^2^* = 0.12) in contraceptive users (Table 2). There were no differences between EFP (93 ± 9 mmHg) and INACTIVE (94 ± 4 mmHg, *p* = 0.838, d = 0.14) or between MLP (93 ± 8 mmHg) and ACTIVE (93 ± 4 mmHg, *p* = 0.949, d = 0.00). In males, there were no differences between visits (99 ± 6 mmHg vs. 99 ± 6 mmHg, *p* = 0.472, d_z_ = 0.16). There was no main effect of group (*p* = 0.254, *n_p_^2^* = 0.18) between males, eumenorrheic women and contraceptive users (Table 3).

#### 3.3.2. Aortic Diastolic Blood Pressure

There were no main effects identified for phase (*n_p_^2^* = 0.00, *n_p_^2^* = 0.21), repeat (*n_p_^2^* = 0.00, *n_p_^2^* = 0.25) or phase × repeat interaction (*n_p_^2^* = 0.04, *n_p_^2^* = 0.06) in eumenorrheic women (Table 1) or contraceptive users (Table 2), respectively. There were no differences between EFP (68 ± 8 mmHg) and INACTIVE (70 ± 3 mmHg, *p* = 0.447, d = 0.33) or between MLP (68 ± 7 mmHg) and ACTIVE (69 ± 4 mmHg, *p* = 0.714, d = 0.18). In males, there were no differences between visits (67 ± 5 mmHg vs. 68 ± 5 mmHg, *p* = 0.383, d_z_ = 0.19). There was no main effect of group (*p* = 0.292, *n_p_^2^* = 0.16) between males, eumenorrheic women and contraceptive users (Table 3).

### 3.4. 17-β-Estradiol and Progesterone

All contraceptive users were confirmed as compliant with pill use. Briefly, 9/12 participants reported 17-β-estradiol concentrations that were below the lower limit of quantification for the plate-based assay (i.e., <8.68 pg/mL) and the remaining 3 participants reported low 17-β-estradiol concentrations (ranging from 8.9–21.6 pg/mL). Additionally, 4/13 eumenorrheic women also demonstrated 17-β-estradiol concentrations that were below the lower limit of quantification in the EFP, with those above the limit of quantification ranging from 9.2 to 24.8 pg/mL, providing a positive confirmation of the menstrual cycle phase. In eumenorrheic women, there were main effects of phase on 17-β-estradiol and progesterone, respectively (*n_p_^2^* = 0.72, *n_p_^2^* = 0.86), with higher concentrations of both hormones in the MLP vs. EFP (Table 1). However, there were no main effects of repeat (*n_p_^2^* = 0.13, *n_p_^2^* = 0.08) or phase × repeat interaction effects (*n_p_^2^* = 0.00, *n_p_^2^* = 0.10). In contraceptive users, there were no main effects of phase (*n_p_^2^* = 0.17, *n_p_^2^* = 0.03), repeat (*n_p_^2^* = 0.03, *n_p_^2^* = 0.21) or phase × repeat interaction (*n_p_^2^* = 0.25, *n_p_^2^* = 0.03, Table 2).

### 3.5. Correlations

**Figure 2:**
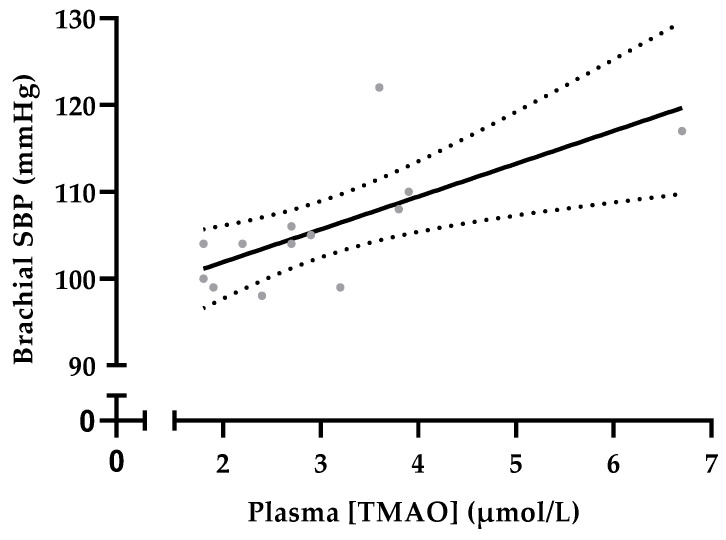
Linear regression model showing the relationship between plasma TMAO concentrations (TMAO) and brachial systolic blood pressure averaged across the menstrual cycle in eumenorrheic women. Data are displayed as the line of best fit with 95% confidence intervals (dotted lines) (r^2^ = 0.489, *p* = 0.008). Individual participants are denoted by grey circles.

There was a significant moderate-to-high positive correlation between TMAO concentration and brachial systolic blood pressure averaged across the menstrual cycle in eumenorrheic women (Pearson’s *r* = 0.705, *p* = 0.007, Figure 2). A linear regression model with which to visualise the relationship between plasma TMAO levels and brachial SBP is shown in Figure 2. No other significant correlations were observed between TMAO and brachial SBP in contraceptive users or males, nor between TMAO and brachial DBP, central SBP or central DBP in any of the three groups.

## 4. Discussion

We report herein that concentration of the gut bacteria-derived cardiometabolic risk marker, TMAO, are consistent across the menstrual cycle in eumenorrheic women, unchanged between pill withdrawal and pill consumption days in combined, monophasic oral contraceptive pill users, and not different between young and healthy, males and females. As expected, brachial systolic blood pressure readings were higher in males than those in age-matched females, but there were no inter-sex differences in brachial diastolic blood pressure or central blood pressure measures derived via arterial tonometry. Lastly, TMAO, blood pressure, 17-β-estradiol and progesterone demonstrated good reproducibility, with no significant differences observed between repeated measures on separate days in men or women.

A recent study assessing serum TMAO levels throughout the menstrual cycle reported no differences between the follicular, ovulatory and luteal phases (~4 μmol/L), and no correlations were observed between TMAO and oestrogen or progesterone [28]. Similarly, we observed no menstrual cycle variability in plasma TMAO, and observed similar absolute TMAO concentrations (~3 μmol/L) in an analogous population. Extending the findings of Bergström and colleagues [28] who excluded women using oral contraceptives, neither did we observed differences in TMAO between pill withdrawal days (i.e., no synthetic hormones ingested) and pill consumption days (i.e., ingestion of synthetic hormones) in healthy, combined, monophasic oral contraceptive pill users, nor did we observe any differences in TMAO between eumenorrheic women and contraceptive users. One of the commonly reported reasons for excluding females from research studies is the need/expectation to conduct experimental testing in a particular phase of the menstrual cycle (which presents a significant time challenge), and/or, due to the use of hormonal contraceptives. Since we and others [28] observed no differences in TMAO levels across the menstrual cycle, or between phases of contraceptive pill use in young females, consideration should be taken to increase the inclusion of these populations in future research studies assessing this gut-derived metabolite, without the need to strictly control the timing of experimental visits.

There are large cyclic fluctuations in concentrations of 17-β-estradiol and progesterone throughout the menstrual cycle which is commonly divided into three phases: the EFP (low concentrations of 17-β-estradiol and progesterone), late follicular phase (high concentrations of 17-β-estradiol and low concentrations of progesterone), and MLP (high concentrations of 17-β-estradiol and progesterone) [26]. Endogenous 17-β-estradiol is considered cardioprotective in premenopausal women and since concentrations of it are higher in the MLP than the EFP, it might be expected that blood pressure would be lower in the MLP. Indeed, some work has shown that brachial blood pressure is higher at the onset of menstruation compared to in later phases in the menstrual cycle [29], but previous research has largely reported no significant differences between phases [30,31,32,33]. In line with the latter observations, we saw no differences in brachial or central blood pressure between the EFP and MLP. A limitation of our study and much of the previous research is that blood pressure was not assessed during the late follicular phase when concentrations of 17-β-estradiol are greatest. Adkisson et al. (2010) reported that brachial and central blood pressure were lowest during this phase [34] and therefore further research may be needed to elucidate the changes in blood pressure throughout the menstrual cycle in premenopausal women.

Owing to the widespread utilisation of hormonal contraceptives amongst young women, assessing their side effects is an important public health consideration. Oral contraceptive pills downregulate endogenous concentrations of 17-β-estradiol and progesterone and have previously been shown to elevate blood pressure in young women in some [19,20] but not all [35] studies. To our knowledge, this is the first study to compare both resting brachial and central blood pressure readings between pill withdrawal and pill consumption days in healthy, normotensive, combined, monophasic oral contraceptive pill users. In line with previous findings in pre-hypertensive contraceptive users using the same pill formulation, we observed no differences in brachial blood pressure between pill withdrawal and pill consumption days [36]. Our data extend these findings and show no differences in central blood pressure between pill days. Moreover, and contrary to some previous research [37], we did not observe any differences in brachial or central blood pressure between oral contraceptive users and eumenorrheic women.

An interesting finding from the current study is that TMAO concentration was not different between similar-aged men and women. This contrasts findings from some previous human studies where men presented with higher TMAO concentration [11,12,13], but aligns with others reporting no inter-sex differences [14,15]. It is known that diet modulates the gut microbiota composition and TMAO concentration [5,38], and sex-specific dietary preferences have been associated with sex differences in TMAO concentration [11]. The consumption of dietary TMAO precursors was not quantified in the present study so it is not possible to deduce whether or not the absence of any differences in TMAO concentrations between the sexes is consequent to similar food preferences. Moreover, we did not quantify Toll-like receptors, which are involved in TMAO-induced platelet activation [39], or the expression of hepatic FMO3, an enzyme which catalyses the rate limiting step in TMAO production [40]. These mechanisms have previously been shown to accelerate TMAO formation in females and have distinct associations with cardiovascular risk.

All participants in the current study were normotensive (*n* = 37) or pre-hypertensive (*n* = 10; one eumenorrheic woman, and nine males). In line with previous observations, brachial systolic blood pressure was higher in males than females [41], but contrary to some reports, we observed no differences in blood pressure between eumenorrheic women and contraceptive pill users. A positive dose–response relationship was previously observed between circulating TMAO levels and hypertension in participants with a high cardiovascular risk [42]. In the current study, a positive correlation was observed between TMAO concentration and brachial systolic blood pressure in eumenorrheic women, but not in females using oral contraceptives or in males. The association between TMAO concentration and cardiovascular risk is typically reported in hypertensive cases and clinical populations [43,44,45]. Thus, it is plausible that this relationship is less frequently observed in cohorts who exhibit low cardiovascular risk.

A secondary aim of this study was to assess the between-day reproducibility of TMAO, blood pressure, 17-β-estradiol and progesterone measures. This study collected samples and corresponding data under basal conditions at the same time of day (±1 h) and following replicated 24 h food intake, allowing the confident assessment and reporting of the consistency of measures within and between individuals over time. Our replicated crossover study allowed, for the first time, the reproducibility between biological repeats to be quantified, and our data suggests that there is good reliability between biological repeats of the aforementioned variables.

## 5. Conclusions

Despite a sex difference in brachial SBP, there were no differences in plasma TMAO concentration and central BP between males and females, throughout the menstrual cycle or between pill withdrawal and pill consumption days in oral contraceptive pill users. Plasma TMAO concentration was positively associated with brachial SBP in eumenorrheic women, but not female pill users or males, and was not correlated with central BP in any of these groups. All measured variables demonstrated acceptable between-day reproducibility. Therefore, while plasma TMAO, female sex hormones and brachial and systolic BP can be reliably determined in young healthy adults, the prognostic value of plasma TMAO concentration for cardiovascular health in these populations requires further research.

## Figures and Tables

**Table 1:** Trimethylamine N-oxide concentrations, peripheral and central blood pressure and sex hormone concentrations in the early follicular phase and mid-luteal phase of the menstrual cycle in eumenorrheic women. Each variable was assessed twice during two separate menstrual cycles.

	Menstrual Cycle Phase				*p*		
	EFP_1_	EFP_2_	MLP_1_	MLP_2_	Phase	Repeat	Interaction
TMAO (μmol/L)	3.0 ± 2.1	2.8 ± 1.8	3.5 ± 2.3	2.9 ± 1.5	0.389	0.368	0.725
Peripheral SBP (mmHg)	106 ± 9	105 ± 9	105 ± 7	106 ± 7	0.892	1.000	0.454
Peripheral DBP (mmHg)	67 ± 8	67 ± 8	67 ± 7	67 ± 7	0.806	0.605	0.885
Central SBP (mmHg)	94 ± 10	92 ± 10	93 ± 9	94 ± 8	0.831	0.640	**0.041**
Central DBP (mmHg)	68 ± 9	67 ± 8	67 ± 8	68 ± 7	0.976	0.917	0.559
17-β-estradiol (pg/mL)	16 ± 9	12 ± 10	48 ± 20	44 ± 35	**<0.001**	0.200	0.963
Progesterone (ng/mL)	1.7 ± 1.2	2.2 ± 2.9	27.3 ± 11.3	23.1 ± 12.8	**<0.001**	0.325	0.275

TMAO, Trimethylamine N-oxide concentrations; EFP_1_, early follicular phase biological repeat 1; EFP_2_, early follicular phase biological repeat 2; MLP_1_, mid-luteal phase biological repeat 1; MLP_2_, mid-luteal phase biological repeat 2; SBP, systolic blood pressure; DBP, diastolic blood pressure. Data presented as mean ± SD. Bold text denotes *p* ≤ 0.05. *n* = 13 for TMAO, peripheral blood pressure and sex hormones 17-β-estradiol and progesterone. *n* = 11 for central blood pressure.

**Table 2:** Trimethylamine N-oxide concentrations, peripheral and central blood pressure and sex hormone concentrations in the INACTIVE and ACTIVE pill phase in combined monophasic oral contraceptive pill users. Each variable was assessed twice during two separate pill cycles.

	Contraceptive Pill Phase				*p*		
	INACTIVE_1_	INACTIVE_2_	ACTIVE_1_	ACTIVE_2_	Phase	Repeat	Interaction
TMAO (μmol/L)	2.3 ± 1.2	4.2 ± 5.6	1.9 ± 0.8	2.8 ± 2.2	0.330	0.127	0.594
Peripheral SBP (mmHg)	109 ± 5	107 ± 5	108 ± 4	109 ± 7	0.406	0.187	0.109
Peripheral DBP (mmHg)	69 ± 4	68 ± 3	68 ± 4	67 ± 4	0.157	0.209	0.818
Central SBP (mmHg)	95 ± 4	93 ± 4	93 ± 4	93 ± 5	0.543	0.268	0.293
Central DBP (mmHg)	71 ± 4	69 ± 4	69 ± 4	68 ± 4	0.161	0.115	0.477
17-β-estradiol (pg/mL)	6.1 ± 7.2	3.6 ± 7.8	3.2 ± 4.7	4.2 ± 5.7	0.158	0.589	0.082
Progesterone (ng/mL)	0.7 ± 0.4	0.7 ± 0.4	0.7 ± 0.3	0.6 ± 0.4	0.601	0.115	0.551

TMAO, Trimethylamine N-oxide concentrations; INACTIVE_1_, no synthetic hormone consumption phase biological repeat 1; INACTIVE_2_, no synthetic hormone consumption phase biological repeat 2; ACTIVE_1_, synthetic hormone consumption phase biological repeat 1; ACTIVE_2_, synthetic hormone consumption phase biological repeat 2; SBP, systolic blood pressure; DBP, diastolic blood pressure. Data presented as mean ± SD. *n* = 12 for TMAO, peripheral blood pressure and sex hormones 17-β-estradiol and progesterone. *n* = 10 for central blood pressure.

**Table 3:** Trimethylamine N-oxide concentrations and peripheral and central blood in eumenorrheic women, combined, monophasic oral contraceptive pill users and males.

	Eumenorrheic Women	Contraceptive Users	Males	*p*
TMAO (μmol/L)	3.0 ± 1.3	2.8 ± 1.4	3.0 ± 1.8	0.514
Peripheral SBP (mmHg)	106 ± 7	108 ± 5	119 ± 7	**<0.001**
Peripheral DBP (mmHg)	67 ± 6	68 ± 3	67 ± 4	0.765
Central SBP (mmHg)	93 ± 9	93 ± 4	99 ± 6	0.254
Central DBP (mmHg)	68 ± 7	69 ± 3	67 ± 4	0.292

TMAO, Trimethylamine N-oxide concentrations; SBP, systolic blood pressure; DBP, diastolic blood pressure. Data presented as mean ± SD. Bold text denotes *p* ≤ 0.05. Data were averaged across the menstrual cycle (early follicular phase and mid-luteal phase) and pill days (inactive and active pill phases) to allow a group comparison between the eumenorrheic women, contraceptive users and males.

## Data Availability

Data can be provided at reasonable request from the corresponding author. The data are not publicly available due to privacy.
